# Combined stereotactic radiation therapy and immunotherapy for metastatic uveal melanoma

**DOI:** 10.3389/fonc.2025.1567504

**Published:** 2025-05-08

**Authors:** Valeria V. Nazarova, Kristina V. Orlova, Zakhra R. Magomedova, Denis S. Romanov, Raman Maskalenka, Andrey A. Yarovoy, Vera A. Yarovaya, Oxana P. Trofimova, Irina Zh. Shubina, Lev V. Demidov

**Affiliations:** ^1^ FSBI “N.N. Blokhin National Medical Research Center of Oncology”, Ministry of Health of the Russian Federation, Moscow, Russia; ^2^ Federal State Autonomous Educational Institution (FSAEI) of Higher Education «N.I. Pirogov Russian National Research Medical University» of the Ministry of Health of the Russian Federation, Moscow, Russia; ^3^ Center for Innovative Medical Technologies, Moscow, Russia; ^4^ Radiation Oncology Center “OncoStop”, Moscow, Russia; ^5^ Svyatoslav Nikolaevich (S.N.) Fedorov National Medical Research Center (NMRC) “Mezhotraslevoy Nauchno-Technichesky Komplex (MNTK) “Eye Microsurgery”, Moscow, Russia

**Keywords:** metastatic uveal melanoma, immunotherapy, stereotactic radiotherapy, overall survival, response rate

## Abstract

**Aim:**

Uveal melanoma (UM) is a rare primary intraocular malignant tumor with an extremely poor prognosis. Our study evaluated the feasibility to improve metastatic UM treatment outcomes with a combined approach of immunotherapy and radiation therapy.

**Methods:**

The retrospective study enrolled 24 patients with metastatic uveal melanoma who had combined treatment with stereotactic radiation therapy (RT) and immune checkpoint inhibitor therapy. 35% of patients received combination immunotherapy, and the others received mono-immunotherapy with anti-PD-1 drugs. All patients underwent stereotactic RT for metastases in the liver (75% patients), bones (8%), soft tissues (8%), brain (4%), and lungs (4%).

**Results:**

Overall response rate (ORR) was 39.1%. Complete response (CR) was achieved in 8.7% patients and partial response (PR) – in 30.4% patients, median progression free survival (PFS) was 11.6 months [95% confidence interval (CI), 5.4-14.4], and median overall survival (OS) was 27.6 months [95% CI, 16.9 - 49.1].

**Conclusions:**

The study has demonstrated a safe combination of stereotactic radiation therapy and immune checkpoint inhibitor immunotherapy in patients with metastatic uveal melanoma. The combination shows a potential treatment option for this patient cohort since no other effective therapies are available at present.

## 1 Introduction

Uveal melanoma (UM) is a primary intraocular malignant tumor developing from melanocytes of the iris (3-5%), ciliary body (5-8%) or choroid (about 90% cases) (1). It is a rare disease with an incidence of 1 case per 100,000 people per year in Europe and 5.3 to 10.9 cases per million people per year worldwide (2). On the other hand, UM is the most common primary malignant intraocular tumor among the adult population. Risk factors for uveal melanoma include fair skin and congenital melanosis (3). Median OS of patients with metastatic UM is approximately 13.4 months, and the 2-year survival rate does not exceed 8% (4). Initially, distant metastases are found in less than 1% of patients at their first visit to an ophthalmologist (5). Within 5 years after treatment of the primary tumor, metastases are detected in 31% of cases, within 15 years - in 45% and within 25 years - in almost 50% of patients (6). The most common sites of metastasis are liver (60.5%), lungs (24.4%), skin/soft tissues (10.9%), and bones (8.4%) (7).

Most studies and retrospective analyses of metastatic UM discuss local methods of treatment (radiofrequency ablation, chemo- or immuno-embolization, and others) for liver metastases, which the authors regard as the main and the only disease manifestations in most patients. Nevertheless, the studies demonstrated that patients who underwent local treatment of liver metastases had an OS advantage as compared to those who had no such treatment.

In a meta-analysis of 2019 including 78 articles and 2494 patients, the median OS of patients with metastatic UM regardless of the therapeutic approach was 1.07 years (range: 0.59–2.50). The median OS of patients after isolated liver perfusion was 1.34 years (hazard ratio (HR) 0.92, 95% CI: 0.87-0.97, p=0.0040), after immunoembolization - 1.63 years (HR 0.97, 95% CI: 0.95-1.00, p=0.0080), after surgical treatment - 1.43 (HR 0.94, 95% CI: 0.92-0.96, p<0.0001), and after immune checkpoint inhibitors - 0.59 years (HR 1.13, 95% CI: 1.06-1.20, p<0.0001) (8). Differences in OS with different treatment options might be explained by the selection of patients on the base of inclusion criteria for one or another type of therapy.

It should be noted that immunotherapy may improve survival rates for patients with metastatic UM compared to that of standard chemotherapy. A Danish population-based study found that median progression-free survival (PFS) of chemotherapy-treated patients (n = 32) was 2.5 months versus 3.5 months in the immunotherapy group receiving anti-PD-1 monotherapy or combination of CTLA-4 with anti-PD-1 (HR 0.43; 95% CI: 0.28–0.67; p < 0.001). One-year OS increased from 25.0% to 41.9% and median OS - from 7.8 months to 10.0 months, respectively (95% CI: 0.34–0.79; p = 0.003) (9).

However, literature review showed that the ORR of anti-PD1 therapy in patients with metastatic uveal melanoma is dramatically low compared to that of patients with metastatic cutaneous melanoma, and the median PFS of the anti-PD-1 immunotherapy in patients with uveal melanoma achieves only 3.8 months (10). The effectiveness of combination immunotherapy in patients with metastatic uveal melanoma is lower than that in patients with metastatic cutaneous melanoma; nevertheless, at present it is one of the major options for UM treatment. A retrospective study of 15 patients with UM reported an ORR of 16.7% and a median PFS of 2.8 months (11). Najjar YG et al. reported a study of combination immunotherapy in 89 patients with UM from 14 centers. The results showed that ORR was 11.6%, median PFS was 2.7 months, and median OS was 15 months (12). In the multicenter phase II GEM1402 trial (NCT02626962) which evaluated the efficacy of combination immunotherapy in 50 treatment-naïve patients, the ORR was 12%, median PFS and OS – 3.3 and 12.7 months, respectively (13). It is interesting to compare the combination immunotherapy results for UM with the effectiveness of combination immunotherapy for cutaneous metastatic melanoma achieved in the multicenter randomized phase III study CheckMate 067: the median OS in the combination immunotherapy group was 72.1 months (95% CI: 38.2 -NE) based on the results of 6.5- year follow-up of patients with a 6.5-year overall survival rate of 49% and an ORR of 58% (14). The difference in the effectiveness between the immunotherapy effectiveness in advanced cutaneous melanoma and metastatic uveal melanoma is obvious and is determined by different biology of these tumors.

Nowadays, immunotherapy is used for treatment of most malignancies with different effectiveness with regard to the tumor type. Immunotherapy resistance and the search for the ways to overcome it have become increasingly important research areas in oncology. Studies of response predictors, drugs with other mechanisms of action (HDAC inhibitors, bispecific antibody - tebentafusp) in combination with anti-PD1+-anti-CTLA-4 immunotherapy, combination with local methods of treatment for local metastatic liver lesions (radiation therapy, percutaneous liver perfusion) have the potential to change poor prognosis for metastatic uveal melanoma.

At the same time, the role of radiation therapy (RT) in patients with advanced forms of tumors has been increasing in the era of immunotherapy. The optimal sequence of RT and immunotherapy generally depends on the immunotherapy type. Apparently, simultaneous administration of anti-PD-1/PDL-1 has the highest synergy with radiation therapy (15).

Metastatic UM still remains a poorly manageable disease, therefore current therapeutic approaches should be modified to achieve therapeutic benefits.

At the N.N. Blokhin National Medical Research Center of Oncology, patients with uveal melanoma with isolated liver metastases received combined treatment: a combination of radiation therapy (radiotherapy/radiosurgery) and immunotherapy with immune checkpoint inhibitors from 2018 to 2024. The paper presents a retrospective analysis of the effectiveness and safety of simultaneous radiotherapy/radiosurgery and ICI immunotherapy.

## 2 Materials and methods

The main criteria for inclusion in the analysis were the following: confirmed diagnosis of uveal melanoma, metastatic and/or inoperable tumor, absence of contraindications to immunotherapy (anti-PD-1, a combination of anti-PD-1 and anti-CTLA-4) and stereotactic radiation therapy (SRT) of distant metastases, and the signed patient’s informed consent. An additional important criterion included measurable metastatic lesions in the liver according to RECIST 1.1 with at least one lesion over 10 mm and less than 30 mm available for stereotactic radiotherapy, LDH ≤ULN (upper level of normal). Other criteria included standard requirements of the treatment safety, in particular, dose-volume restrictions for healthy organs and tissues in case of stereotactic radiotherapy.

The main exclusion criteria were indications for surgical treatment with time to the appearance of metastases >5 years or an option for R0 resection; more than 3 metastatic lesions in one organ and a total of more than 5 lesions; patients with severe concomitant diseases or life-threatening acute complications of the underlying disease; concomitant diseases and/or conditions that significantly increased the risk of developing adverse events during the study; and required therapy with glucocorticoids or any other drugs with immunosuppressive effects within 14 days before randomization.

The primary endpoint for evaluation of the therapy effectiveness was ORR, which was calculated as the proportion of patients with complete or partial response, and local control (LC) of irradiated lesions (ORRil and LCil), which was calculated as the proportion of patients with complete or partial response or stabilization of the irradiated lesions (according to RECIST 1.1). Secondary endpoints of the therapy effectiveness included PFS and OS. The Kaplan–Meier method with indication of median survival was used to analyze PFS and OS. PFS was calculated from the date of therapy initiation, and the event was recorded in case if disease progression or death were registered. The safety endpoints were the total rate of adverse events (AEs) of any grade and AEs of grades 3–4 calculated as the proportion of patients with grade 3–4 AEs in the whole number of patients (evaluated according to NCI CTC AE 5.0).

All patients received anti-PD-1 immunotherapy or a combination of anti-PD-1 and anti-CTLA-4 therapy and stereotactic radiotherapy before or after the start of systemic therapy in compliance with all requirements for high-tech radiation therapy. Stereotactic radiotherapy (SRT) was selectively delivered to 1–3 lesions, prioritizing the largest tumor foci situated in anatomically favorable locations to optimize therapeutic precision and mitigate radiation-related toxicity. Treatment was performed on linear electron accelerators Varian Clinac 2100–2300 iX (Varian) and 3-dimensional conformal radiation therapy (3D-CRT) was used; if there were advantages in terms of dose distribution and radiation exposure to critical organs, IMRT (intensity modulated radiation therapy) and VMAT (volume intensity modulated arc therapy) dose delivery technologies were used; MRI data with intravenous contrast and PET-CT were used to choose the volume of radiation; respiratory movement control technology was used in patients with irradiation of liver metastases, in particular, holding the breath at inhale under the control of the RPM (Real-time Position Management); optimal fixation devices (such as thermoplastic masks, vacuum mattresses, position boards) were used for positioning the patients.

The statistical analysis and visualization of the obtained data were conducted using the Python lifelines library. Descriptive statistics for quantitative variables is presented as mean (± standard deviation, SD) and median (1st and 3rd quartiles), and as absolute and relative frequencies for qualitative variables. Survival analysis was conducted using the Kaplan-Meier method, and the Cox proportional hazards regression model was used to estimate hazard ratios (HRs) for various covariates.

The study was conducted in accordance with the Declaration of Helsinki and Good Clinical Practice guidelines. All patients provided written informed consent. The study was reviewed and approved by the Institutional Review Board of the FSBI “N.N. Blokhin National Medical Research Center of Oncology” of the Ministry of Health of the Russian Federation with the approval number: #11 of 5 February, 2018.

## 3 Results

The retrospective analysis included 24 patients who referred to the N.N. Blokhin National Medical Research Center of Oncology from 2018 to 2024.

One patient was excluded from the analysis due to multiple metastatic liver lesions diagnosed prior to RT. Twenty four patients underwent stereotactic radiation therapy to the largest lesions, three patients during the first 6 months of immunotherapy, other patients at the start of ICI therapy, or immediately after the first infusion of the drug. 23 of them received anti-PD-1 immunotherapy or a combination of anti-PD-1 and anti-CTLA-4 therapy, one patient had no any drug therapy due to a severe polyvalent allergic reaction of anaphylactic shock caused by drug injection, and therefore the patient was excluded from the analysis of the effectiveness of radiation therapy with immunotherapy.

**Table 1** presents major patients’ characteristics. The analysis evaluated 24 patients with metastatic uveal melanoma including 18 women (75%) and 6 men (25%). The median age at the start of anti-metastatic therapy was 58.7years (from 36 to 76 years). Seventeen (71%) patients underwent enucleation as part of treatment of the primary tumor; other 7 (29%) patients received organ-preserving treatment - brachytherapy or transpupillary thermotherapy. Response to treatment was evaluated at weeks 9–12 after the start of systemic therapy according to RECIST 1.1 criteria on the base of the results of the abdominal MRI with i.v. contrast (Primovist or Omniscan) and PET-CT/CT results.

**Table 1:** Characteristics of the patients included in the analysis.

Number of patients	n=24
Age at the start of treatment for metastatic uveal melanoma, years	58.7 (36-76)
Sex, n (%)	
male	6 (25%)
female	18 (75%)
ECOG	
0	12 (50%)
1	12 (50%)
Therapy of primary tumor	
enucleation	17 (71%)
organ-preserving methods	7 (29%)
Time from initial diagnosis to registration of distant metastases median (range), years	2 (0–19 years)
<3	13 (54%)
3-5	7 (29%)
>5	4 (17%)
Irradiated metastatic lesions	
liver	18 (75%)
soft tissues	2 (8%)
bones	2 (8%)
brain	1 (4%)
lung	1 (4%)
Line of therapy	
1	18 (75%)
2	5 (21%)
3 and >	1 (4%)
Immunotherapy	23 (96%)

Primary tumor samples of two patients were analyzed in a prognostic molecular genetic study. One tumor had a deletion of a copy of the short arm of chromosome 3 and increased copy number of the short arm of chromosome 8 (8q). Both tumors had mutation c.626A>T (p.Gln209Leu) in exon 5 of the GNA11 gene [COSMIC ID 52969], and the period to the registration of metastases reached 12 months. PCR tests of tumor samples of other 2 patients revealed driver mutations in the GNAQ gene. The data in **Table 1** demonstrate that most patients (83%) developed distant metastases within 5 years after treatment of primary uveal melanoma. The molecular genetic characteristics identified in the tumors suggested unfavorable prognosis for those patients.

**Table 2** presents the detailed drug therapy regimens, targets, doses of stereotactic radiotherapy and response.

**Table 2:** Results of the combined stereotactic radiation therapy and immunotherapy.

N	Age (yr)	Time from diagnosis to first metastases registration	ECOG	Line of therapy	Drug therapy	Site of irradiation	Dosage RT SFD/ TFD	LC in the irradiated site	Best response
1	67	3 yrs	1	1	Nivolumab 240 mg	liver	18/54 Gy	PR	PR
2	55	3 yrs	1	2 (1- ChT)	Nivolumab 240 mg	liver (2 foci)	15/45 Gy	PR	PR
3	67	19 yrs	1	1	Nivolumab 1 mg/kg + ipilimumab 3 mg/kg, then Nivolumab 480 mg	bones and liver	6-7/30–35 Gy and 15/45 Gy	CR	CR
4	76	1 yr	1	1	Nivolumab 240 mg	liver	15/45 Gy	PR	PD
5	51	1 yr	0	2 (1- ChT)	Nivolumab 1 mg/kg + ipilimumab 3 mg/kg, then Nivolumab 480 mg	liver (3 foci)	15/45 Gy	PR	S
6	65	8 mos	1	1	Pembrolizumab 200 mg	liver	18/54 Gy	S	PD
7	68	1 yr	1	2 (Pembrolizumab 200 mg)	Nivolumab 1 mg/kg + ipilimumab 3 mg/kg, then Nivolumab 480 mg	liver (2 foci)	15/45 Gy	PR	PR
8	73	17 yrs	1	2 (Tebentafusp)	Nivolumab 3 mg/kg	liver	10/50 Gy	PR	PD
9	66	3 yrs	0	1	Prolgolimab 1 mg/kg	liver	15/45 Gy	PR	PR
10	53	2 yrs	0	1	Nivolumab 1 mg/kg + ipilimumab 3 mg/kg, then Nivolumab 240 mg	liver	15/45 Gy	PR	S
11	61	1 yr	1	3 (1-Pembrolizumab 200 mg + vorinosta; 2 – ChT)	Pembrolizumab 200 mg	soft body tissues (2 foci)	8/40 Gy	S	S
12	36	3 mon	0	1	Nivolumab 1 mg/kg + ipilimumab 3 mg/kg, then Nivolumab 240 mg	liver	15/45 Gy	S	S
13	53	4 mon	0	2 (Pembrolizumab 200 mg)	Nivolumab 1 mg/kg + ipilimumab 3 mg/kg, then Nivolumab 240 mg	Soft body tissues (2 foci)	8/40 Gy	PR	S
14	38	0	0	1	Pembrolizumab 200 mg	brain	9/27 Gy	PR	PR
15	60	4 mon	0	1	Nivolumab 1 mg/kg + ipilimumab 3 mg/kg, then Nivolumab 240 mg	liver	15/45 Gy	CR	CR
16	67	6 yr	1	1	Pembrolizumab 200 mg	liver	10/50 Gy	PR	PR
17	54	10 yr	0	1	Pembrolizumab 200 mg	lung	12/60 Gy	PR	PR
18	55	4 yr	0	1	Prolgolimab 1 mg/kg	liver	15/45 Gy	S	S
19	63	2 yr	1	1	Prolgolimab 1 mg/kg	liver	17/51 Gy	PD	PD
20	55	2 yr	0	1	Prolgolimab 1 mg/kg	liver	15/45 Gy	S	S
21	56	4 yr	0	1	Prolgolimab 1 mg/kg	bone	15/45 Gy	S	S
22	38	3 yr	0	1	Nivolumab 1 mg/kg + ipilimumab 3 mg/kg, then Nivolumab 240 mg	liver	15/45 Gy	S	S
23	71	3 yr	1	1	Prolgolimab 1 mg/kg	liver	8/24 Gy	PD	PD
24*	61	2 yrs	1	1	none (allergic reaction)	liver (2 foci)	16/48 Gy	S	S

*excluded from the analysis.

PD, progressive disease; PR, partial response; CR, complete response; S, stabilization; + the response at the time of analysis; RT, radiation therapy; SFD, single focal dose; TFD, total focal dose; LC, local control; TTP, time to progression; mos, months; yr, year; Gy, Gray; ChT, chemotherapy.

The ORR of the combined therapy accounted for 39.1% (n=9), two patients had a complete response (8.7%), and seven patients had partial responses (30.4%). At the same time, the ORRil accounted for 60.9%, and the local control rate of irradiated lesions reached 91.3% (n=21).

**Table 3** presents the detailed outcomes of the combined therapy.

**Table 3:** Overall response rate in the irradiated lesions.

Response	In the irradiated lesion		Overall response	
	n	%	n	%
	n	%	n	%
ORRil/ORR (CR & PR)	14	60.9	9	39.1
CR	2	8.7	2	8.7
PR	12	52.2	7	30.4
SD	7	30.4	9	39.1
PD	2	8.7	5	21.7

ORRil, overall response rate of irradiated lesions; ORR, overall response rate; CR, complete response; PR, partial response; SD, stable disease; PD, progressive disease.

**Figure 1:**
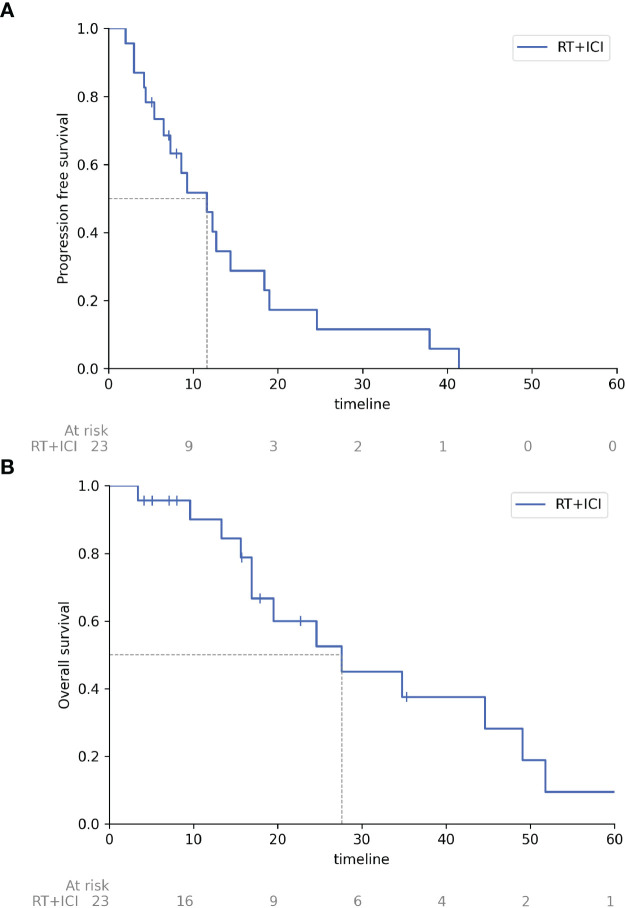
Progression free survival and overall survival of patients receiving combined therapy with radiation therapy and immune checkpoint inhibitors. **(A)** Progression free survival, **(B)** Overall survival. RT, radiation therapy; ICI, immune checkpoint inhibitors, timeline, months.

**Figure 1** demonstrates the results of combined therapy with RT and ICI evaluated by Kaplan – Meier: median progression-free survival achieved 11.6 months (95% CI 5.4-14.4) and median overall survival – 27.6 months (95% CI 16.9 - 49.1).

**Figure 2:**
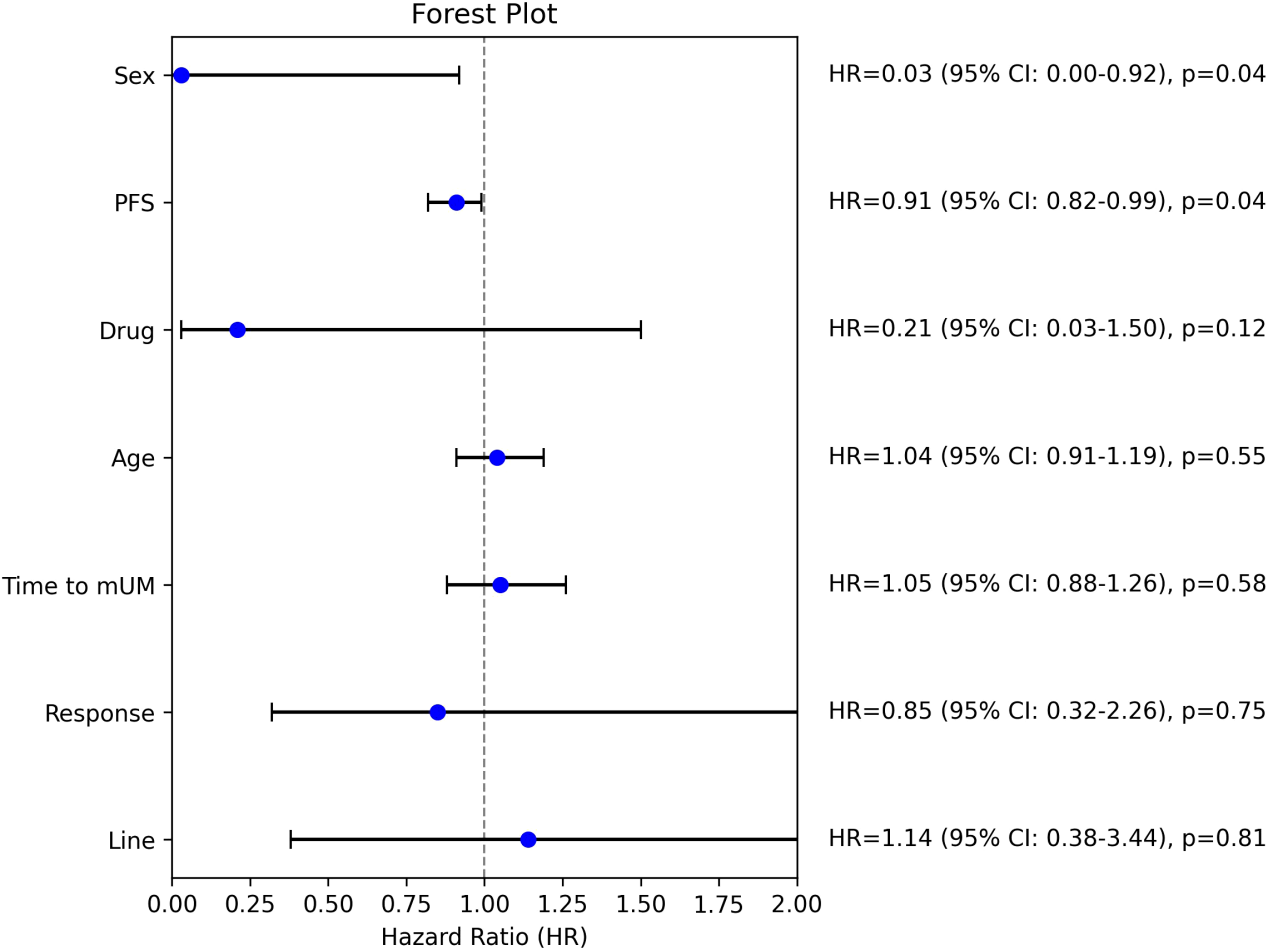
Subgroup analysis; HR, hazard ratio, mUM, metastatic uveal melanoma, PFS, progression free survival.

The subgroup analysis (**Figure 2**) using the Cox proportional hazards regression model revealed that sex (HR: 0.03, 95% CI: 0.00–0.92, p=0.04) and progression free survival (PFS) (HR: 0.91, 95% CI: 0.82–0.99, p=0.04) were statistically significant predictors of overall survival. Female sex and longer PFS were associated with a substantially lower risk of death. The type of therapy (monotherapy vs. combination therapy) showed a trend toward improved outcomes after combination therapy (HR: 0.21, 95% CI: 0.03–1.50, p=0.12), but that did not reach statistical significance.

Interestingly, the time to the development of metastatic uveal melanoma (HR: 1.05, 95% CI: 0.88–1.26, p=0.58) was not significantly associated with overall survival. The lack of significance might be explained by the inclusion of patients with a favorable prognosis (disease-free interval >5 years) only in cases where surgical intervention was not feasible, potentially introducing selection bias.

Adverse events were noted in 91% (n=21) of patients in the course of therapy, in particular: most often general weakness of grade 1-2 (91%), autoimmune hepatitis of grade 1-2 (22%), hypothyroidism (22%), pain syndrome associated with the installation of a tracer or radiation therapy (30%), individual increase of transaminases grade 3, most likely associated with the installation of a tracer (13%), and adrenal failure (4%). Adverse events of grade 3 in two patients were immune-mediated arthritis and hepatitis, which required steroid treatment (methylprednisolone at a dose of 1 mg/kg), and considering severe AEs, the therapy was discontinued or suspended.

## 4 Discussion

According to the literature, systemic therapy regimen leads to the median PFS and median OS of mono-immunotherapy of 2.6 and 7.6 months (16); combination immunotherapy - 3.3 and 12.7 months (13); local treatment – 9.1 and 17.1 months (transarterial chemoembolization, TACE) and 4.3 and 17.1 (isolated liver chemoperfusion) (17). The best results in the reported studies were achieved after surgical removal of metastases in patients with a favorable prognosis: median OS was 25 months for R0 resection and 16 months for R1/2 resection (18). Our results, such as median PFS of 11.6 months (95% CI 5.4-14.4) and median OS of 27.6 months (95% CI 16.9 - 49.1) significantly exceeded PFS and OS of the systemic therapy only or local therapies and are comparable to OS after R0 resection of metastases in patients with a favorable prognosis. It is important to note that the majority of patients in our study (83%) had an unfavorable prognosis, taking into account the development of metastases after treatment of the primary tumor. The only publication similar to our study in its major findings is the paper of Grynberg S, 2022 (19), which presented the results of a retrospective study evaluating the effectiveness of a combination of radiation therapy and immunotherapy for metastatic uveal melanoma and demonstrated the advantage of combined therapy (RT + immunotherapy). The difference of that study was the use of external beam radiotherapy (EBRT), whereas our study always used stereotactic radiotherapy. Grynberg S included 38 patients with metastatic uveal melanoma in the study; 50% of patients received anti-PD-1 therapy and 50% received combined anti-PD-1 and anti-CTLA-4 therapy. Only 9 patients received EBRT or stereotactic radiotherapy during immunotherapy (group A); 29 patients received only immunotherapy (group B). ORR accounted for 44% in group A versus 10% in group B (p = 0.004). Median PFS was 22 months in group A versus 3 months in group B (HR = 0.37, p = 0.036). Median overall survival was also higher in group A, 26 months versus 7.5 months in group B (HR = 0.34, p = 0.03). Toxicity was comparable in both groups.

Noteworthy, the patients who received a combination of radiation therapy and immunotherapy had the high rates of PFS and OS: median PFS was 22 months in Grynberg’s study, and 11.6 months (95% CI 5.4-14.4) in our study; median OS - 26 months and 27.6 months (95% CI 16.9 - 49.1), respectively. The combined approach resulted in ORR of 44% in Grynberg’s study and 39.1% in our study, and, most importantly, these results were the highest ORR and OS among all treatment options for metastatic uveal melanoma. Interestingly, despite similarities of the study reported by Grynberg et al. and our study, the PFS were different. The detailed analysis suggested possible explanations for the inconsistent results, such as similar ORR 44 and 39.1% and OS 26 and 27.6 months but different disease progression rates 33 and 21.7%, respectively. We noted similar periods of PFS (22 months) and OS (26 months) in Grynberg et al. study. The similarity of the periods might imply that: 1) patients either had no specific antitumor therapy after recording disease progression (iCPD, immune-confirmed progressive disease) or it was ineffective, or 2) disease progression was registered when tumor burden and clinically advanced disease suggested no any opportunity for further effective treatment. On the other hand, that was associated with the choice of iRECIST for evaluation the therapy effectiveness, while our choice was RECIST 1.1. Our choice was determined by a number of reasons. Firstly, iRECIST was registered in the Russian Federation for monitoring the effectiveness of immunotherapy only in 2021; therefore, since 2018 we made clinical decisions regarding the majority of patients included in the study on the base of RECIST 1.1. Secondly, we initially did not expect a 91% local control of the irradiated lesions and planned to evaluate this parameter, as well (and possibly, to analyze the relationship between the local and systemic response to therapy). It is well known that iRECIST criteria in relation to irradiated lesions is irrelevant, therefore RECIST 1.1 was a universal evaluation system for all manifestations of the disease, regardless of the volume of therapy applied to the lesions. Although RECIST 1.1 was the base for clinical decisions, the majority of patients of our study, even those with disease progression, were monitored for 1-1.5 months to avoid untimely change of therapy line. The only exceptions were patients with oligoprogression, i.e. having one or individually growing lesions, who received stereotactic radiotherapy to those lesions and immunotherapy according to the previous regimen. These patients, despite the registered disease progression, which corresponded to the recorded occurring event on the survival curve, did not progress further for a long time though the systemic therapy was not changed, and that status could no longer affect the PFS rate, but determined high OS rates. Grynberg et al. had no discussion about treatment or lack of treatment of patients after the onset of iCPD. However, in our study most patients, after recognition of the ineffectiveness of the immunotherapy, received other antitumor therapies such as chemotherapy (with taxanes and platinum drugs), targeted therapy (trametinib), re-challenge of the immunotherapy (anti-PD-1 inhibitors), their combinations (lenvatinib and pembrolizumab), external beam radiation therapy, and liver-directed therapy (radiofrequency ablation (RFA)), isolated liver chemoperfusion).

The number of patients receiving combination immunotherapy may also alter the survival rates. Our study included only 35% (8/23) patients receiving the combination of ipilimumab and nivolumab (IpiNivo) at the start of radiation therapy (i.e., at inclusion in the study), while Grynberg’s study included 78% (7/9) patients with such type of therapy. We cannot have a conclusion about the role of an anti-CTLA4 inhibitor added to an anti-PD-1 inhibitor in this context (in fact, it is difficult to draw conclusions on the base of two retrospective analyses of the treatment of 9 and 23 patients, respectively), however, we would like to mention the following observations in this regard. First, the median PFS differed between patients receiving monotherapy and combination immunotherapy in our study: 8.6 months (95% CI: 3.0–14.4) versus 12.7 months (95% CI: 6.5–19.0), with an HR of 0.71 (95% CI: 0.27–1.85; p=0.48). Similarly, the median OS was 24.6 months (95% CI: 13.3–44.6) for monotherapy and 51.8 months (95% CI: 9.6–NE) for combination therapy, with an HR of 0.25 (95% CI: 0.05–1.17; p=0.08). Although these differences were not statistically significant, the results suggested a trend toward improved OS in patients treated with combination therapy.

The second observation results from a different perspective of view on the relationship between ORR and disease progression, since there is another parameter which is undoubtedly acceptable for oncologists: stabilization of the disease (SD). Thus, speaking about the best result achieved, stabilization of the disease as well as a complete or partial response reflects the systemic therapy effect. Grynberg et al. reported that the proportion of patients with tumor progression (i.e., not stabilization) who received combination immunotherapy with radiation therapy was 43% and without RT - 55%; and in case of mono-immunotherapy without RT, it increased up to 78%. However, the data on mono-immunotherapy in combination with radiation therapy were irrelevant since this group of therapy included only two patients with stable disease (i.e., their ORR and PD rates showed zero at analysis). All patients of our study receiving the IpiNivo combination had the best response different from progressive disease, while only 73% (11/15) of patients had the same response in the anti-PD-1 immunotherapy group.

Therefore, we may suggest that a combination of anti-CTLA4 and anti-PD1 inhibitors combined with stereotactic radiotherapy can lead to better results compared to other therapeutic options. On the other hand, however, there is an option of using ipilimumab in the second line therapy after disease progression, which is formally absent at the start of the combination immunotherapy.

Though a combined approach of immunotherapy and radiation therapy seems to have a good potential, so far the statistics of the reported research has been obviously insufficient to manage the abscopal effect efficiently. The single and total focal doses of radiation therapy have not been determined, yet; most studies, though, indicate single doses of 6 to10 Gy, but other authors suggest achieving the abscopal effect with standard fractioning, moderate hypofractioning, hyperfractioning, and high doses of stereotactic radiotherapy or radiosurgery.

The order of radiotherapy and immunotherapy is not absolutely clear; taking into account real experience and literature data, we could assume that radiation therapy performed on the nearest date to the start of immunotherapy reaches the best effectiveness, however, others may consider the therapies order unimportant or prefer to conduct radiation therapy within some weeks before or after the start of systemic antitumor therapy.

The criteria for selecting metastases appropriate for irradiation are still being discussed; logic and general consideration suggests that they should present foci of an average size of 3–5 cm with a localization that is safe regarding the risk of radiation damage.

The discussion of a possible development of the abscopal effect involves a number of unresolved issues. For instance, could we achieve the same abscopal effect in case of irradiation of either intracranial, visceral or bone metastases? Which approach of optimal immunotherapy (anti-PD1, anti-PD-L1 or anti-CTLA-4) would lead to the most frequent abscopal effect?

At present, the combination of various local treatment methods and immunotherapy is being actively studied. Preliminary results have been published in a phase II clinical trial (NCT03472586) evaluating the efficacy of combining immunotherapy with liver immunoembolization, as well as in the CHOPIN trial (NCT04283890), a phase I/II randomized single-center study, and SCANDIUM II (NCT04463368), a phase I randomized multicenter trial that investigated the combination of immunotherapy with percutaneous and isolated hepatic perfusion. The growing interest in such studies highlights the significance of a combined approach for treating patients with metastatic uveal melanoma.

The presented study has demonstrated a safe combination of stereotactic radiation therapy and immune checkpoint inhibitor immunotherapy in patients with metastatic uveal melanoma. This combination shows promise as a potential therapeutic option for this patient cohort since no other effective therapies are available at present.

## Data Availability

The original contributions presented in the study are included in the article/Supplementary Material. Further inquiries can be directed to the corresponding author.
